# Breast cancer care compared with clinical Guidelines: an observational study in France

**DOI:** 10.1186/1471-2458-11-45

**Published:** 2011-01-20

**Authors:** Marie Lebeau, Simone Mathoulin-Pélissier, Carine Bellera, Christine Tunon-de-Lara, Alain Daban, Francis Lipinski, Dominique Jaubert, Pierre Ingrand, Virginie Migeot

**Affiliations:** 1 Pôle de Cancérologie, Hématologie et Pathologie Tissulaire, Service de Radiothérapie, CHU de Poitiers, France; 2 Réseau de Cancérologie d'Aquitaine, 229 cours de l'Argonne, 33076 Bordeaux Cedex, France; 3 Unité de Recherche et d'Épidémiologie Cliniques, CRLCC Institut Bergonié, 229 cours de l'Argonne 33076 Bordeaux Cedex, France; 4 Inserm CIC-EC7, 126 rue Léo Saignat, 33076 Bordeaux Cedex, France; 5 Université Victor Segalen Bordeaux 2, 146 rue Léo Saignat 33076 Bordeaux Cedex, France; 6 Service de Chirurgie, CRLCC Institut Bergonié, 229 cours de l'Argonne, 33076 Bordeaux Cedex, France; 7 Réseau de Cancérologie de Poitou-Charentes, Poitiers, France; 8 Pôle de Cancérologie, Hématologie et Pathologie Tissulaire, Service de Radiothérapie, CHU de Poitiers, France; 9 Centre Oncoradiothérapie de la Côte Basque, Bayonne, France

## Abstract

**Background:**

Great variability in breast cancer (BC) treatment practices according to patient, tumour or organisation of care characteristics has been reported but the relation between these factors is not well known. In two French regions, we measured compliance with Clinical Practice Guidelines for non-metastatic BC care management and identified factors associated with non-compliance at clinical and organisational levels.

**Methods:**

Eligible patients had invasive unilateral BC without distant metastases and at least two contacts with one of the two regional healthcare systems (2003-2004) in the first year after diagnosis. Medical data were collected from patient medical records in all public and private hospitals (99 hospitals).

The care process was defined by 20 criteria: clinical decisions for treatment and therapeutic procedures. Each criterion was classified according to level of compliance ("Compliant", "Justifiable" and "Not Compliant") and factors of non-compliance were identified (mixed effect logistic regression).

**Results:**

926 women were included. Non-compliance with clinical decisions for treatment was associated with older patient age (OR 2.1; 95%CI: 1.3-3.6) and region (OR 3.0; 95%CI: 1.2-7.4). Non-compliance with clinical decisions for radiotherapy was associated with lymph node involvement or the presence of peritumoural vascular invasion (OR 1.5; 95%CI: 1.01-2.3) and non-compliance with overall treatment (clinical decisions for treatment + therapeutic procedures) was associated with the presence of positive lymph nodes (OR 2.0; 95%CI: 1.2-3.3), grade III versus grade I (OR 2.9; 95%CI: 1.4-6.2), and one region of care versus another (OR 3.5; 95%CI: 1.7-7.1). Finally, heterogeneity of compliance in overall treatment sequence was identified between local cancer units (p < 0.05).

**Conclusion:**

This study provides interesting insights into factors of non-compliance in non-metastatic BC management and could lead to quality care improvements.

## Background

The treatment of breast cancer is complex and requires multidisciplinary care [1,2]. To deal with this complexity and reduce the number of inappropriate interventions, government agencies and specialist societies have developed Clinical Practice Guidelines (CPGs). Nevertheless, many studies [3-5] have highlighted a great variability of practices in breast cancer treatment according to patient or tumour characteristics, and the way care is organised. Other studies in the literature emphasise organisational factors as determinants of compliance with CPGs [6-11]. In 2003, all French regions began to develop local cancer units (LCUs) dedicated to cancer care. The aim at this time was to improve the quality of care by enhancing the quality of therapeutic multidisciplinary committee discussions. These multidisciplinary committees base their decisions on national or regional CPGs in order to harmonise practices. Our objectives in this study were firstly to measure the compliance with CPGs for the management of non-metastatic breast cancer care and secondly to identify factors associated with non-compliance at a clinical and organisational level (LCU).

## Methods

### Study design and population selection

Eligible patients had a pathological diagnosis of invasive unilateral breast cancer without distant metastases (from June 2003 to October 2004), at least two contacts with one of the two regional healthcare systems between diagnosis and their first year of follow-up, no previous malignancy, and provided written informed consent. These eligible patients were reported by public and private hospitals in the two regions (additional file 1: 99 eligible hospitals: 13% with less than 11 BC surgeries per year and 51% with more than 50). All oncologists in both regions reported every patient with a first diagnosis of invasive non-metastatic breast cancer. Following this, a letter explaining the aims of the study was sent to each patient, together with a consent form and a questionnaire collecting personal information. Once the consent form had been received, medical data was collected from the patient's medical record. The logistics of data collection were carried out by a experienced research team specifically dedicated to the project (research assistants and research practitioners). Full details of the present design were published earlier [12,13]. Clinical data covered the care process from diagnosis to the last sequence of treatment (follow-up was excluded). The care process was divided into six potential steps: initial and complementary surgery, breast or chest radiotherapy, lymph node radiotherapy, chemotherapy, and hormone therapy. This study was approved by the ethics committee.

### Evaluation of compliance with practice guidelines

We categorised the available non-metastatic BC management pathways from French national CPGs (additional file 2) into 20 criteria (Additional file 3). These criteria were used to assess compliance with the care process for each patient according to the six steps defined. Each criterion was classified into three levels of compliance: (C) compliance with CPGs; (J) justifiable non-compliance, i.e., not strictly compliant but documented justification due to the patient's general status, preference or a change during the course of care management (for example, chemotherapy interruption related to adverse effects) or other factors; (NC) non-compliance with CPGs and no justification available in the patient's medical record. For each criterion, the conditions needed to define three-level compliance were defined by a specific questionnaire sent to the relevant experts (surgeon, pathologist, radiologist, chemotherapist and radiation oncologist); moreover, these definitions were agreed upon by each expert. For example, we defined the type of breast and lymph node surgery recommended for different tumour sizes (< 1 cm, 1-3 cm, and >3 cm). Finally, the entire expert group reached a consensus on the description of criteria with particular attention to the justifiable category.

### Study variables

To measure the appropriateness of medical decisions, we also defined compliance with criteria which included therapeutic decisions as opposed to diagnostic or organisational elements (criteria 1, 2, 6, 15, 18, 20). Moreover, we studied both the clinical decisions for treatment and therapeutic procedures of the least compliant therapeutic steps (criteria 6-14). In addition to assessing the 20 criteria, overall treatment compliance (clinical decisions for treatment and therapeutic procedures) with CPGs was determined for each patient. This depended on the compliance with criteria 1-20 of clinical care. Because we modelled the probability of non-compliance, the overall treatment sequence compliance was coded '0' if all therapeutic clinical decisions for treatment and therapeutic procedures were compliant (C) or justifiable (J), and '1' if at least one of these was not compliant with standards (NC).

Explanatory variables were selected from the literature review and related to the patient and her social status [1,14,15], to the tumour [6,7,15-18] and to the healthcare system [1,6-8]. Patients' characteristics were analysed by age (< 50, 50-69, ≥70), educational level (less than high-school diploma, at least high-school diploma), cohabitation status (living alone, living with other(s)), menopausal status (pre- or postmenopausal). Tumour characteristics were analysed by localisation (central, medial/lateral, several quadrants), nodal status (negative, positive), histological tumour size (≤10 mm, 11-30 mm, >30 mm), hormonal receptor status (oestrogen or progesterone receptors positive, both receptors negative), lymph node and peritumoural vascular invasion (presence/absence), histological grade (I, II, III). Tumour staging was recorded according to the American Joint Committee on Cancer classification [19]. Variables related to the healthcare system were sector of care (public, private, both), surgical hospital status (teaching, non-teaching) and region where patients underwent surgery. Given that variations in care across practices has been documented previously in France [20], we suspected that non-compliance could vary across the LCUs (15 Units) and thus considered this variable when evaluating non-compliance with CPGs.

### Statistical analysis

We examined factors associated with non-compliance with CPGs. Aside from the LCU, all variables were first fitted in univariate logistic regression models. Variables significant in the univariate analyses (p < 0.20), as well as relevant clinical variables, were then fitted in a classical multivariate logistic model [21]. This process was independently repeated for each outcome variable: non-compliance regarding 1) clinical decision for treatment, 2) radiotherapy, 3) overall treatment sequence.

As the compliance of many patients at the same LCU could depend on local management practices, we expected data clustering to occur at the level of the LCU. We thus relied on a mixed-effect model by including a random effect in our classical multivariate model [22]. By doing so, the LCUs were assumed to give rise to another source of random variation in addition to the residual variation left unexplained by the fixed-effect variables. The resulting mixed-effect model accommodated for correlations within LCUs, or similarly, heterogeneity of non-compliance between LCUs. We tested this heterogeneity of practices across LCUs by comparing the mixed-effect model to the initial classical logistic model using the likelihood ratio test (LRT) [21]. We reported odds ratios (OR) with their 95% confidence intervals (95% CI) and performed all statistical analyses with the SAS 8.2 software.

## Results

### Population

Informed consent was obtained from 955 of the 1416 eligible patients; other patients were refusals (193, 13%) or non-response (269, 18%). Twenty nine patients were excluded from the analysis for the following reasons: other non-breast cancer (n = 1), bilateral tumour (n = 21) or medical record not available (n = 7). Mean patient age was 58.5 years (range: 24-89). Patients, tumour characteristics and care sector itinerary are presented in Table 1. All but nine patients underwent surgery: 688 (75%) had conservative surgery, 226 (25%) had mastectomy and three patients had lymph node surgery without breast surgery. Sentinel node biopsy was performed in 214 patients (26%) and lymph node surgery in 842 (91%). A breast or chest radiotherapy was used for 902 patients (97%) and all but three procedures of conservative surgery were followed by radiotherapy. Lymph node radiotherapy was performed on axillary lymph nodes in 52 (6%) patients, on internal mammary lymph nodes in 507 (55%) and on supraclavicular lymph nodes in 520 (56%) patients. Chemotherapy was administered to 444 (48%) patients, among whom 21% participated in a clinical trial, and 670 (72%) patients received hormonal therapy, including nine who participated in a clinical trial. Finally, four of ten patients were managed in both the private and public sector, and surgery was mostly performed in non-teaching hospitals.

**Table 1:** Description of breast cancer population (926 patients)

Characteristics	N	(%)
Age (years)		
< 50	250	(27)
50-69	468	(51)
≥ 70	208	(22)
Educational level		
Less than baccalaureate (secondary-school diploma)	627	(71)
Baccalaureate or higher	262	(29)
Live alone		
No	738	(79)
Yes	194	(21)
Tumour localisation		
Central	47	(5)
Several quadrants	247	(27)
Medial or lateral	603	(68)
Stage AJCC/UICC*		
Stage 0 and 1	447	(49)
Stage 2	332	(36)
Stage 3	137	(15)
Menopausal status		
Postmenopausal	590	(67)
Premenopausal	284	(33)
Nodal status		
Negative	566	(63)
Positive	336	(37)
Histological size (mm)		
≤ 10	250	(29)
10-30	544	(62)
> 30	76	(9)
Hormonal receptor status		
At least one receptor positive	742	(81)
Two receptors negative	170	(19)
Peritumoural vascular invasion		
No	385	(42)
Yes	229	(25)
Unknown	312	(34)
Histological grade		
Grade I	211	(24)
Grade II	422	(48)
Grade III	254	(72)
Care sector itinerary		
Public	333	(36)
Private	231	(25)
Both (public and private)	362	(39)
Status of surgical hospital		
Teaching	223	(24)
Not teaching	703	(76)
Region of surgery		
Region 1	558	(63)
Region 2	326	(37)

*AJCC: American Joint Committee on Cancer

### Compliance by care management step

The surgical procedures were compliant to CPGs (criteria 1-5) for 65% (604) of patients; the radiotherapy procedures (criteria 6-14) for 48% (442); chemotherapy procedures (criteria 15-17) for 73% (679); hormonal therapy procedures (criteria 18-19) for 88% (816); and the multidisciplinary committee discussion (criterion 20) for 59% (546) of patients. Compliance with treatment criteria in each step showed that the time to radiotherapy was the least compliant (Table 2) of criteria. Nine patients (1%) had no surgery. For 107 (12%) patients, no indication of the tumour size was found, so compliance of the initial surgical decision was coded as 'J'. Conservative surgery showed a higher rate of compliance than mastectomy (72% vs 47%). Clinical decisions for radiotherapy mostly complied with CGPs following conservative surgery (99%) and mastectomy (86%). When chemoterapy was indicated, the vast majority of patients received it (99%), but chemotherapy was also given to 29% of patients who had no clinical decision according to the criterion. Hormonal therapy was given to 3% of patients for whom receptors were negative and not given to 11% of patients for whom receptors were positive.

**Table 2:** Criteria by step of breast cancer care according to the presence of compliance with Clinical Practices Guidelines (C), justifiable non-compliance (J) or non-compliance (NC) (926 patients)

	Compliant		Justifiable		Non-compliant		Total*
	N	(%)	N	(%)	N	(%)	
Surgery							
Clinical decision							
Initial	672	(73)	229	(25)	25	(3)	926
Complementary	712	(77)	66	(7)	148	(16)	926
Procedure							
≥ 10 lymph nodes	602	(71)	117	(14)	123	(15)	842
Complete resection	716	(78)	_	_	198	(22)	914
Surgical staging	903	(99)	_	_	11	(1)	914
Radiotherapy							
Clinical decision							
Breast or chest	892	(96)	0	(0)	34	(4)	926
Axillary	880	(95)	2	(0)	44	(5)	926
Internal mammary	625	(67)	196	(22)	105	(11)	926
Supraclavicular	627	(68)	165	(18)	134	(14)	926
Procedure							
Breast or chest	696	(77)	165	(18)	41	(5)	902
Axillary	38	(73)	8	(15)	6	(11)	52
Internal mammary	376	(74)	111	(22)	20	(4)	507
Supraclavicular	382	(73)	113	(22)	25	(5)	520
Time to radiotherapy	568	(62)	30	(3)	313	(34)	911
Chemotherapy							
Clinical decision	550	(59)	235	(25)	141	(15)	926
Procedure							
Protocols/cycles	373	(84)	15	(3)	56	(13)	444
Time to chemotherapy	355	(80)	33	(7)	56	(13)	444
Hormonal therapy							
Clinical decision	822	(89)	19	(2)	85	(9)	926
Procedure	616	(92)	29	(4)	25	(4)	670
Multidisciplinary meeting	526	(57)	20	(2)	380	(41)	926

* All patients were concerned by clinical decision criteria. With regard to therapeutic procedure criteria, only patients whose clinical decision gave rise to a therapeutic procedure were concerned.

### Factors associated with treatment compliance

With regard to *clinical decisions for treatment*, the rate of non-compliance with CPGs was 71% (657 patients). Variables selected from the univariate analyses and first included in a multivariate fixed-effect model were: age (p = 0.001), education level (p = 0.03), cohabitation status (p = 0.06), stage (p = 0.18), hormonal status (p = 0.06), care sector (p < 0.0001), teaching status of surgical hospital (p = 0.0004) and region where patients underwent surgery (p < 0.0001). After fitting the multivariate fixed-effect model, three factors were associated positively with non-compliance (p < 0.05): age, status of surgical hospital, and region where patients underwent surgery. Introducing the LCUs as a random effect appeared to decrease the residual variability (LRT, p < 0.05), which again indicated heterogeneity in compliance with *clinical decisions for treatment*, across LCUs and emphasised the need to keep this variable in our model. The mixed-effect model suggested that compared to patients under 50 years of age, patients over 70 years had a two-fold risk of non-compliant care management (OR = 2.1; 95% CI, 1.3-3.6). Similarly, there was a three-fold increase in the risk of non-compliance for one region compared to the second one (OR = 3.0; 95% CI, 1.2-7.4). Finally, note that although the teaching status of the surgical hospital had a significant effect in the fixed-effect model (p = 0.0004), it was no longer significant in the mixed-effect model (p = 0.12).

Of all the therapeutic steps considered, *radiotherapy *was the least compliant step: we observed at least one non-compliant clinical decision for radiotherapy or radiotherapy procedure criterion for 484 patients (52%). Variables selected from the univariate analyses and first included in a multivariate fixed-effect model were: tumour localisation (p = 0.036), nodal status (p = 0.009), histological size (p = 0.018), hormonal receptor status (p = 0.007), peritumoural vascular invasion (p = 0.001), grade (p = 0.015), and region where patients underwent surgery (p = 0.04). Peritumoural vascular invasion, histological grade and region were positively associated with non-compliance in the multivariate fixed-effect model. Including the LCU as a random effect decreased the residual variability (LRT, p < 0.05), suggesting the presence of heterogeneity of compliance in radiotherapy across LCUs. Based on this mixed-effect model, only the presence of peritumoural vascular invasion remained significant at the 5% level, and it was associated with 1.5-fold risk of non-compliance (OR = 1.5; 95% CI, 1.01-2.3).

Within the overall *treatment sequence *(clinical decision treatment and therapeutic procedure), at least one criterion was not completely compliant with CPGs in 88% of 926 patients. Variables selected from the univariate analyses and first included in the multivariate fixed-effect model were: care sector (public or private) (p < 0.0001), region where patients underwent surgery (p < 0.0001), grade (p = 0.0002), stage (p = 0.0003), nodal status (p = 0.0004), hormonal receptor status (p = 0.01), peritumoural vascular invasion (p = 0.02), histological size (p = 0.02), age (p = 0.06), educational level (p = 0.06), cohabitation status (p = 0.14), and teaching status of surgical hospital (p = 0.20). After adjustment, three factors were positively associated with non-compliance in the overall treatment sequence in the multivariate model (p < 0.05, Table 3): nodal status, histological grade, and region where patients underwent surgery. Finally, introducing the LCU as a random effect significantly decreased the residual variability (LRT, p < 0.05), suggesting the presence of heterogeneity of compliance in overall treatment sequence across LCUs and reinforcing the need to keep this variable in our model. Estimates from this mixed-effect model suggested that, compared to patients without lymph node metastasis, lymph node-positive patients had a two-fold risk of non-compliance (OR = 2.0; 95% CI, 1.2-3.3); patients with grade III tumours had a three-fold risk of non-compliance compared to patients with grade I (OR = 2.9; 95% CI, 1.4-6.2); and patients whose surgery was performed in one of the two regions had a 3.5-fold higher risk than the other region (OR = 3.5; 95% CI, 1.7 -7.1).

**Table 3:** Multivariate analyses for non-compliance of overall treatment sequence, therapeutic indications and radiotherapy (926 patients)

	Compliance (C/J)		Non-compliance (NC)		Logistic model		Logistic mixed model	
	
Variables	No of patients	%	No of patients	%	OR	95% CI	OR	95% CI
Clinical decisions for treatment (n = 856)								
Age (years)								
< 50	82	(33)	168	(67)				
50 - 69	148	(32)	320	(68)	1.0	0.7-1.4	1.1	0.8-1.7
> 70	39	(19)	169	(81)	1.9	1.2-3.0	2.1	1.3-3.6
Surgical hospital								
Teaching	86	(39)	137	(61)				
Not teaching	183	(26)	520	(74)	1.4	1.02-2.0	1.7	0.9-3.3
Region								
Region 1	199	(36)	359	(64)				
Region 2	62	(19)	264	(81)	2.1	1.5-3.0	3.0	1.2-7.4
Radiotherapy (clinical decision and procedure) (n = 817)								
Peritumoural vascular invasion								
No	211	(55)	174	(45)				
Yes	97	(42)	132	(58)	1.6	1.1-2.3	1.5	1.01-2.3
Unknown	134	(43)	178	(57)	1.4	0.99-1.9	1.3	0.9-1.9
Histological grade								
Grade I	113	(54)	98	(46)				
Grade II	211	(50)	211	(50)	1.0	0.7-1.4	1.0	0.7-1.5
Grade III	104	(41)	150	(59)	1.6	1.1-2.3	1.5	0.97-2.4
Region								
Region 1	285	(51)	273	(49)				
Region 2	143	(44)	183	(56)	1.5	1.1-2.0	1.3	0.7-2.3
Overall treatment sequence (n = 805)								
Nodal status								
Negative	88	(16)	478	(84)				
Positive	26	(8)	310	(92)	2.0	1.2-3.2	2.0	1.2-3.3
Histological grade								
Grade I	33	(16)	178	(84)				
Grade II	65	(15)	357	(85)	0.9	0.5-1.4	0.9	0.5-1.5
Grade III	15	(6)	239	(94)	2.9	1.5-5.7	2.9	1.4-6.2
Region								
Region 1	90	(16)	468	(84)				
Region 2	22	(7)	304	(93)	3.5	1.9-5.2	3.5	1.7-7.1
								

## Discussion

This study showed three main results: BC compliance, factors associated with non-compliance and a multidisciplinary and multi-hospital organisation to BC care which explains the variability in BC practices. Together these results can be used to enhance BC care.

The first results of our study concern BC care compliance that ranged from 88% for hormonal therapy to 48% for radiotherapy, essentially due to the non respect of delay to radiotherapy. With respect to overall treatment sequences, the management of non-metastatic breast cancer was fully compliant (20 criteria) with CPGs in only 12% of cases. This proportion increased to 29% when only clinical decisions for treatment steps were considered. To our knowledge, this is the first time that results of overall BC therapeutic care with details on procedures and clinical decision sequences have been reported in a large BC population. Indeed, most recent publications have only focused on single therapeutic care management step (surgery, radiotherapy or chemotherapy) according to BC stage and most do not provide details on overall compliance with clinical decisions. International studies [1,3,6,17,23-26] have found a higher compliance rate for performing a biopsy before surgery, radiotherapy and chemotherapy, and a lower rate for performing radiotherapy after conservative breast surgery and hormonal therapy. However, caution is required with such comparisons because many studies did not accurately explain the compliance criteria they used. Two other French regional studies have compared breast cancer management with CPG recommendations and found approximately similar results. The first study (100 patients by audits of medical records) [27] reported the same proportion of non-compliance for clinical decision for lymph node surgery and for clinical decision for radiotherapy. The second [5] observed strict compliance with clinical decision for treatment in 1995: 92-96% for surgery, 71-85% for chemotherapy, 72-93% for radiotherapy and 83-94% for hormonal therapy. Our results show similar or higher levels of compliance.

We also reported in our study factors related to BC non-compliance. Older age and region were factors to explain non-compliant clinical decisions for treatment and confirm then results reported previously [3,15]. Concerning radiotherapy, the least compliant step, non-compliance was associated with three prognostic factors (nodal status, peritumoural vascular invasion and histological grade) and the region of surgical treatment. Finally, we showed an association between overall non-compliance and the presence of positive lymph nodes, histological grade III and region. Lymph node involvement and histological grade are known prognostic factors for breast cancer recurrence and survival. These two clinical characteristics are part of CGPs for breast cancer management and are considered, for example, in making decisions regarding the need for chemotherapy or radiotherapy. Our findings emphasise their impact on overall non-compliance. Concerning the region of BC treatment, a factor associated with non-compliance, the region with a higher compliance rate had already implemented breast cancer CPGs (2004) by local specialist involvement in regional guidelines at the time of the study. This implementation could be explained partially by the regional differences. But some disparity for care accessibility (equipment or personnel resources) between the two regions could explain part of these differences, as reported by others [28]. The more recent publications of factors related to the implementation of CGPs [9-11,29,30] showed that patient factors, such as comorbidities or very short life expectancy, reduce the chance that guidelines are followed because they do not encourage the physician to prescribe aggressive therapy. Physician factors related to implementation could be seniority, lack of awareness and limited agreement with guidelines. Moreover, organisation factors such as limited time, work pressure and limited support from peers have been described as barriers to change. Our results showed finally a variability in compliance with CPGs that may be due to the LCU. This discrepancy in practices underlines the need to enhance cancer care coordination and multidisciplinary care at a local level, so that variation in care can be reduced. Whether we modelled the non-compliance of practices within the overall treatment sequence, with regard to clinical decision for treatment, or with regard to radiotherapy only, introducing the LCU as a random variable significantly decreased residual variability. These results confirm the presence of heterogeneity across LCUs, or equivalently, the presence of correlation within LCUs.

This study has a number of strengths and limitations. Firstly, concerning the population, data collection and definition of non-compliance, we cannot exclude a population selection bias since a proportion of women were not included (refusal or non response) and we included probably more patients from hospitals with high volumes (but regional data are available for comparison concerning all stages of cancer, metastatic and non-metastatic BC). We proposed 20 criteria that distinguish clinical decisions for treatment from the procedures resulting from the decision. Such analysis of compliance is necessary to understand better the complexity of the care process, particularly in BC management. A recent synthesis [9] showed that the complexity of CPGs can be one factor of non-compliance and could so explain a low rate of overall compliance. Our results showed that compliance was lowest for radiotherapy lead time and multidisciplinary approach. Long radiotherapy lead times have already been reported in Italy [31] and in a critical review of the literature [28] and are partly due to lack of equipment or human resources [32]. The French national Cancer Plan has attempted to respond to this shortcoming [33]. Patient management in multidisciplinary meetings is recommended for every cancer patient, but this recommendation was too recent (2003) at the date of our study and may explain the relatively low compliance rate of 57%.

We grouped compliance (C) and justifiable non-compliance (J) in the same category, particurlaly to ensure the homogeneity of the data within the non-compliance category (NC). Another statistical choice could be an analysis with three levels of compliance (a polychotomous regression). However, our primary interest was to specifically assess determinants of non-compliance/non conformity to CPGs, without distinguishing compliant and justifiable decisions, which we considered as equivalent. Indeed, from a practical point of view, it is best to specifically target those factors associated with non-compliance so that actions can be undertaken. In view of these objectives, logistic regression using two levels was the optimal modelling approach.

Another potential limitation of our results was the availablity of data in medical records to explain non-compliance and our choice of professionals or organisational factors. We could not collect all data on every professional (for instance, experience or age) but BC management is multidisciplinary and several specialists are involved in medical decisions at each BC management step. Concerning organisational factors, we cannot relate overall compliance to the hospital's volume since the breast cancer care steps are often in different hospitals (surgery in one hospital and radiotherapy in another...) and these hospitals may have different volumes. It therefore seemed that the most interesting data was the LCU. However, we use hospital volume in a recent publication focused on surgery step [12].

We must underline several points concerning the interpretation of our results. Firstly, a negative impact on outcomes of high non-compliance rate with CPGs for non-metastatic breast cancer management should be interpreted carefully. Each criterion received the same weight whatever its prognostic value and some patients had more non-compliant criteria than others. However, more than two thirds of the patients had fewer than three non-compliant criteria. A specific consensus [34] amongst local breast cancer experts will be required to develop and apply criteria integrating weights according to prognostic impact. This definition of non-compliance of care management could increase heterogeneity between LCUs, even though variations in practice between LCUs are not surprising since similar variations have been reported for larger institutions [35]. In addition, it should be noted that service improvements for local and regional cancer care organisations (including implementation of LCU) was probably too recent at the date of this study to have an homogeneous impact on medical practices. It would be useful to reproduce this study in another cohort of patients to ascertain if compliance with CPGs has improved over time. Currently, the implementation of the regional guidelines cannot be used as the only factor to explain a better compliance in one region. Indeed, it is well known that adherence with these guidelines depends on many other factors [9-11] such as guideline, professional, patient and environmental characteristics. Our study was implemented between 2003 and 2004, and we studied compliance based on CPGs implemented at this time and in these areas.

Secondly, in the presence of heterogeneity across LCUs, it is important to rely on an appropriate statistical model; otherwise misleading conclusions can be derived. We relied on a mixed-effect model which allowed us to account for the presence of clusters. Other approaches have been suggested to test for the heterogeneity of responses in the context of binary variables, such as the estimation of the median odds ratio as suggested recently [36]. Applying this method did not modify our initial conclusions with respect to heterogeneity across practices.

Finally, this study provides interesting insights into factors of non-compliance in non-metastatic BC management and could lead to quality care improvements. It must be followed by feedback to LCUs and to the regional organisation. Feedback is considered as one of the most efficient methods for improving the application of CPGs [37]. Repeated studies will be required to confirm the likely impact of regional service improvement initiatives over time.

## Conclusion

Our findings emphasise also the need for the national scientific and local cancer care organisations to provide further clarification of BC clinical practice guidelines (which may currently be too complex for a complete implementation in local cancer units). For decision makers and national health care authorities, priorities need to be defined in the quality of cancer care regarding care coordination and local cancer units as well as their equipment and personnel resources.

## Authors' contributions

ML participated in the design of the study/survey, collected and analysed data, drafted the manuscript; SMP designed the study, supervised collection, data analyses and drafted the manuscript. CB assisted with the statistical analysis and helped to draft the manuscript. CTL, AD, FL, DJ, PI were involved in designing the research question, final results and manuscript drafts. VM designed the study, supervised collection, and drafted the manuscript. All authors read and approved the final manuscript.

The REPERES cohort was funded by the PHRC (Hospital Program for Clinical Research from the French Health Ministry) and the INSERM (French National Institute of Health and Medical Research). The funding sources had no involvement in the study.

## Conflict of interest

The authors declare that they have no competing interests.

## Pre-publication history

The pre-publication history for this paper can be accessed here:

http://www.biomedcentral.com/1471-2458/11/45/prepub

## Supplementary Material

Additional file 1 **Breast cancer surgery volume per year according to number hospitals and patients in REPERES study (data available from a database managed by the French Ministry of Health)**. Distribution of patients according to four categories of volume of breast cancer surgery hospital: 10 and less/11-50/51-150/151 and over (data of volume surgery were provided by administrative data, years 2003 and 2004). Click here for file

Additional file 2 **National and international Clinical Practice Guidelines for the management of non-metastatic breast cancer published before 2004 (non exhaustive list of Guidelines, except in France)**. a non exhaustive list of national and international Clinical Practice Guidelines for the management of non-metastatic breast cancer. Click here for file

Additional file 3 **Definition of compliance by criterion in cancer care process for non-metastatic invasive breast cancer according to: compliant (C), justifiable (J), not compliant (NC)**. List of 20 criteria of Breast Cancer management pathways. These criteria were used to assess compliance with the care process for each patient according to the six steps defined. Each criterion was classified into three levels of compliance: (C) compliance with CPGs; (J) justifiable non-compliance, i.e., not strictly compliant but documented justification due to the patient's general status, preference or a change during the course of care management (for example, chemotherapy interruption related to adverse effects) or other factors; (NC) non-compliance with Clinical Practice Guidelines and no justification available in the patient's medical record. Click here for file
