# Supplementary material for: Breast cancer care compared with clinical Guidelines: an observational study in France
Source: BMC Public Health. 2011 Jan 20;11:45. doi: 10.1186/1471-2458-11-45 (PMC3037311; doi:10.1186/1471-2458-11-45)
Supplement: Additional file 1 — Breast cancer surgery volume per year according to number hospitals and patients in REPERES study (data available from a database managed by the French Ministry of Health). Distribution of patients according to four categories of volume of breast cancer surgery hospital: 10 and less/11-50/51-150/151 and over (data of volume surgery were provided by administrative data, years 2003 and 2004). [file 1471-2458-11-45-S1.DOC]

**Additional file 1: Breast cancer surgery volume per year according to number hospitals and patients in REPERES study (data available from a database managed by the French Ministry of Health)**

| Surgery volume per year | Number of hospitals (%)  (regional administrative data : mean of 2003 and 2004) | Number of patients (%)  REPERES study |
| --- | --- | --- |
| 10 and less | 13 (13) | 19 ( 2) |
| 11-50 | 35 (36) | 137 (15) |
| 51-150 | 29 (29) | 414 (44) |
| 151 and over | 22 (22) | 362 (39) |
