# Supplementary material for: Breast cancer care compared with clinical Guidelines: an observational study in France
Source: BMC Public Health. 2011 Jan 20;11:45. doi: 10.1186/1471-2458-11-45 (PMC3037311; doi:10.1186/1471-2458-11-45)
Supplement: Additional file 2 — National and international Clinical Practice Guidelines for the management of non-metastatic breast cancer published before 2004 (non exhaustive list of Guidelines, except in France). a non exhaustive list of national and international Clinical Practice Guidelines for the management of non-metastatic breast cancer. [file 1471-2458-11-45-S2.DOC]

**Additional file 2: National and international Clinical Practice Guidelines for the management of non-metastatic breast cancer published before 2004 (non exhaustive list of Guidelines, except in France)**

| **Year** | **CPG** | **Society** | **Internet link** |
| --- | --- | --- | --- |
| **France** | | | |
| **2004** | Recommandations pour la prise en charge des cancers du sein non métastatiques | Réseau de Cancérologie d'Aquitaine (RCA) | <http://sircamt.canceraquitaine.org/rca> |
| **2002** | Chirurgie des lésions mammaires: prise en charge de première intention | Agence Nationale d'Accréditation et d'Évaluation en Santé (ANAES) | <http://www.has-sante.fr/> |
| **2001** | Cancers du sein non métastatiques – Standards Options et Recommandations | Fédération nationale des centres de lutte contre le cancer (FNCLCC) | <http://www.sor-cancer.fr/> |
| **1998** | Le cancer du sein: recommandations pour la pratique clinique | Agence Nationale d'Accréditation et d'Evaluation en Santé (ANAES) | <http://www.has-sante.fr/> |
| **Canada** | | | |
| **2000** | Guide de pratique clinique pour la prise en charge et le traitement du cancer du sein | Canadian Medical Association (CMA) | <http://www.collectionscanada.gc.ca/eppp-archive/> |
| **United Kingdom** | | | |
| **2002** | Improving outcomes in breast cancer – Manual update | National Health Service (NHS) – National Institute for Clinical Excellence (NICE) | <http://www.nice.org.uk/> |
| **1998** | Guidelines for surgeons in the management of symptomatic breast disease in the united kingdom | British Association of Surgical Oncology (BASO)  Association of Breast Surgery (ABS) | [http://www.baso.org.uk](http://www.baso.org.uk/) |
| **United States** | | | |
| **2000** | Practice guidelines for the treatment of breast cancer | National Comprehensive Cancer Network (NCCN) | [http://www.nccn.org](http://www.nccn.org/) |
| **Italy** | | | |
| **2001** | Practice guidelines for the diagnosis and treatment of breast cancer | Italian FONCAM Guidelines | <http://www.senologia.it/foncam/> |
