# Supplementary material for: Breast cancer care compared with clinical Guidelines: an observational study in France
Source: BMC Public Health. 2011 Jan 20;11:45. doi: 10.1186/1471-2458-11-45 (PMC3037311; doi:10.1186/1471-2458-11-45)
Supplement: Additional file 3 — Definition of compliance by criterion in cancer care process for non-metastatic invasive breast cancer according to: compliant (C), justifiable (J), not compliant (NC). List of 20 criteria of Breast Cancer management pathways. These criteria were used to assess compliance with the care process for each patient according to the six steps defined. Each criterion was classified into three levels of compliance: (C) compliance with CPGs; (J) justifiable non-compliance, i.e., not strictly compliant but documented justification due to the patient's general status, preference or a change during the course of care management (for example, chemotherapy interruption related to adverse effects) or other factors; (NC) non-compliance with Clinical Practice Guidelines and no justification available in the patient's medical record. [file 1471-2458-11-45-S3.DOC]

Additional file 3 . Definition of compliance by criterion in cancer care process for non-metastatic invasive breast cancer according to: compliant (C), justifiable (J), not compliant (NC)

| **Process** |  | **Criteria** | **Compliance** | **Conditions** | **Missing data (MD): number and explanation for choosing justifiable** |
| --- | --- | --- | --- | --- | --- |
| **Surgical indication** | 1 | Initial surgery | **C** | Subclinical tumour and [conservative] or[ total] surgery with axillary surgery  Tumour < 3 cm and [conservative surgery with axillary surgery] or [mastectomy if tumour is central]  Tumour > 3 cm and [mastectomy] or [conservative surgery] with neoadjuvant treatment and axillary surgery |  |
|  |  |  | **J** | Subclinical tumour and [conservative surgery without axillary surgery] or [axillary surgery initially and conservative surgery secondarily]  Tumour < 3 cm and [axillary surgery initially and conservative surgery secondarily] or [mastectomy if tumour is not central]  Tumour > 3 cm and conservative surgery without neoadjuvant treatment and axillary surgery  For every size : no surgery and patient refusal  No data available in the medical record concerning tumor size | 107 MD: they were classified as 'justified' because the lack of such data does not necessarily mean that it had not been taken into account in the management |
|  |  |  | **NC** | Tumour < 3 cm and [conservative surgery or mastectomy if tumour is not central and no axillary surgery] or [axillary surgery without tumour surgery]  Tumour > 3 cm and [axillary surgery initially and conservative surgery secondarily] or [conservative surgery initially and axillary surgery secondarily without neoadjuvant treatment]  For every size : no surgery |  |
|  | 2 | Complementary surgery | **C** | No complete resection  Or axillary surgery if subclinical tumour with surgery tumour initially |  |
|  |  |  | **J** | Axillary surgery if no subclinical tumour with surgery tumour initially  Or tumour surgery if complete resection initially |  |
|  |  |  | **NC** | No complete resection  Or conservative surgery and axillary surgery initially |  |
| **Surgical procedure** | 3 | Number of lymph nodes | **C** | At least 10 axillary lymph nodes in the surgical sample |  |
|  |  |  | **J** | Between 8 or 9 axillary lymph nodes in the sample |  |
|  |  |  | **NC** | Less than 8 axillary lymph nodes in the sample |  |
|  | 4 | Surgical margins | **C** | “complete resection” noted in pathology report |  |
|  |  |  | **J** | Missing data | No MD |
|  |  |  | **NC** | No “complete resection” noted in pathology report |  |
|  | 5 | Surgical stage | **C** | Surgical stage in medical record or at least histological size |  |
|  |  |  | **J** | - |  |
|  |  |  | **NC** | Neither size nor histological stage | 11 MD: such data must appear in medical record |
| **Radiotherapy indication** | 6 | Breast or chest | **C** | Radiotherapy after [conservative surgery] or [after mastectomy if patient presents at least one risk factor or if there was no surgery]  Or no radiotherapy after mastectomy without risk factor |  |
|  |  |  | **J** | Missing data concerning risk factor or patient refusal | No MD |
|  |  |  | **NC** | Other situations |  |
|  | 7 | Axillary lymph nodes | **C** | If no axillary surgery |  |
|  |  |  | **J** | If axillary surgery and more than 10 lymph nodes positives and discussion in multidisciplinary committee |  |
|  |  |  | **NC** | Other situations |  |
|  | 8 | Internal mammary lymph nodes | **C** | Radiotherapy if [no axillary surgery] or [positives nodes] or [tumour central / medial]  Or no radiotherapy if axillary surgery and negatives nodes and tumour neither central nor medial |  |
|  |  |  | **J** | No radiotherapy of internal mammary lymph nodes whereas its indicate but radiotherapy of breast and of supraclavicular lymph nodes  Or missing data concerning the tumoral localisation or concerning nodes status | 165 MD: they were classified as 'justified' because the lack of such data does not necessarily mean that it had not been taken into account in the management |
|  |  |  | **NC** | Other situations |  |
|  | 9 | Supraclavicular lymph nodes | **C** | Radiotherapy if [no axillary surgery] or [positives nodes] or [tumour central / medial]  Or no radiotherapy if axillary surgery and negative nodes and tumour neither central nor medial |  |
|  |  |  | **J** | Missing data concerning the tumoural localisation or concerning node status | Same as criteria#8 |
|  |  |  | **NC** | Other situations |  |
| **Radiotherapy procedure** | 10 | Breast or chest | **C** | 25 to 33 fractions, doses : 45 to 66 Gy |  |
|  | 11  12  13 | Axillary lymph nodes  Internal mammary lymph nodes  Supraclavicular lymph nodes | **J** | 5 to 8 fractions and doses < 40 Gy  20 to 25 fractions and doses < 55 gy  Fractions > 33 and doses > 66 Gy if exclusive radiotherapy  Patient refusal or side effect  Missing data for fractions or for doses | For all radiotherapy procedure (criteria 10,11,12 and 13), altogether, there were 15 records with missing data for fractions or for doses. They were classified as 'justified' because the lack of such data does not necessarily mean that it had not been taken into account in the management |
|  |  |  | **NC** | Other situations |  |
|  | 14 | Times | **C** | If chemotherapy : less than 3 weeks after chemotherapy  If no chemotherapy : less than 8 weeks after surgery  No delay if exclusive or neoadjuvant radiotherapy |  |
|  |  |  | **J** | Missing data concerning delays or type of radiotherapy | 30 MD; they were classified as 'justified' because the lack of such data does not necessarily mean that it had not been taken into account in the management |
|  |  |  | **NC** | Other situations |  |
| **Chemotherapy indication** | 15 | Chemotherapy | **C** | Chemotherapy if [positive nodes] or [negative nodes and at least two risk factors]  Or no chemotherapy if negative nodes and no risk factor |  |
|  |  |  | **J** | No chemotherapy or chemotherapy and one risk factor  Or missing data concerning risk factor | No MD |
|  |  |  | **NC** | Other situations |  |
| **Chemotherapy procedure** | 16 | Chemotherapy | **C** | Protocol: FEC 100 or FEC < 100 and negative nodes or patient older than 70 or EPITAX or FEC 100 then TAXOTERE  Cycles: 4 to 6 if negative nodes, 6 if positive nodes, up to 8 if neoadjuvant  Or patient in therapeutic trial |  |
|  |  |  | **J** | Patient refusal or side effects  Or missing data  Or 5 cycles | 15MD: they were classified as 'justified' because the lack of such data does not necessarily mean that it had not been taken into account in the management |
|  |  |  | **NC** | Other situations |  |
|  | 17 | Times | **C** | Less than 6 weeks after surgery  Or exclusive or neoadjuvant chemotherapy |  |
|  |  |  | **J** | Missing data for delays | 33 MD: they were classified as 'justified' because the lack of such data does not necessarily mean that it had not been taken into account in the management |
|  |  |  | **NC** | Other situations |  |
| **Hormonal therapy indication** | 18 | Hormonal therapy | **C** | Hormonal therapy if hormonal receptors are positive  No hormonal therapy if hormonal receptors are negative |  |
|  |  |  | **J** | Missing data | 19 MD: they were classified as 'justified' because the lack of such data does not necessarily mean that it had not been taken into account in the management |
|  |  |  | **NC** | Hormonal therapy if hormonal receptors are negative  No hormonal therapy if hormonal receptors are positive |  |
| **Hormonal therapy treatment** | 19 | Hormonal therapy | **C** | Post-menopause: tamoxifen or antiaromatase  Peri- or pre-menopause: tamoxifen |  |
|  |  |  | **J** | Missing data | 29 MD: they were classified as 'justified' because the lack of such data does not necessarily mean that it had not been taken into account in the management |
|  |  |  | **NC** | Other situations |  |
| **Multidisciplinary committee** | 20 | Multidisciplinary discussion | **C** | Committee before neoadjuvant treatment or after surgery if axillary radiotherapy or at least once in care management |  |
|  |  |  | **J** | At least one discussion between professionals |  |
|  |  |  | **NC** | Other situations |  |
